# A Sensitive and Versatile Thickness Determination Method Based on Non-Inflection Terahertz Property Fitting

**DOI:** 10.3390/s19194118

**Published:** 2019-09-23

**Authors:** Xuequan Chen, Emma Pickwell-MacPherson

**Affiliations:** 1 Department of Electronic Engineering, The Chinese University of Hong Kong, Hong Kong 999077, China; xqchen@link.cuhk.edu.hk; 2 Physics Department, Warwick University, Coventry CV4 7AL, UK

**Keywords:** thin-film characterization, thickness uncertainty, inflection point, exponential function, fitting

## Abstract

The accuracy of thin-film characterization in terahertz spectroscopy is mainly set by the thickness uncertainty. Physical thickness measurement has limited accuracy for thin-film samples thinner than a few hundreds of micrometers and is sometimes even impossible. The temporal resolution of time-domain terahertz spectrometers is not sufficient to resolve such thin films. Previously reported numerical methods mainly only work for materials with low dispersion and absorption. Here, we propose a novel method for thickness determination by fitting a non-inflection offset exponential function to the material optical properties. Theoretical analysis predicts the best fitting to only be achieved when the correct thickness is given. Transmission measurements on a thin-film polymer, water, and a lactose pallet verify the theory and show the accurate thickness determination and property characterization on materials which are either achromatic or dispersive, transparent or absorptive, featureless or resonant. The measurements demonstrate the best versatility and sensitivity compared to the state-of-art. The method could be widely adapted to various types of research and industrial applications.

## 1. Introduction

Characterizing sample optical properties by terahertz spectroscopy usually requires a sample thickness known beforehand [1]. For bulk and solid samples, the thickness can be conveniently measured by a caliper down to hundreds of micrometers. When time-domain terahertz spectroscopy is applied, the thickness can also be determined from the multiple reflections if the sufficient time-delay could be provided by the thickness and the absorption does not fully vanish the reflections [2]. However, these methods are not applicable for thin-film samples. Physical thickness measurement could not provide sufficient accuracy when the sample thickness is less than few hundreds of micrometers, and it is sometimes inapplicable when the sample is fragile or soft, or in a liquid form. When the sample thickness is beyond the temporal resolution of the time-domain signal, the multiple reflections are closely stacked to result in a single pulse, providing no extra information for thickness extraction. The thickness uncertainty is the major error source in thin-film sample characterization [3].

Fortunately, a physical feature can be used to assist the thickness determination. The optical properties of most materials are relatively flat in the absence of molecular resonance [4,5,6,7,8], while the sample properties extracted with an incorrect thickness shows periodic Fabry-Perot (FP) oscillations in the frequency-domain. A material property with a FP profile does not match our knowledge from many previous studies and thus it is expected to be wrong due to the incorrect given thickness. Several numerical methods have been proposed by iteratively trying different thickness values until the oscillation profile reaches minimum [2,9,10]. The oscillations were evaluated by means of total variation (TV, referred to as the TV approach hereinafter), which sums up the difference of all neighboring refractive index *n* and/or extinction ratio *κ* values. The TV approach is simple and fast but with limited accuracy and versatility. The evaluation requires the variation contributed by the FP oscillations to be obviously larger than the variation of the sample properties, thus the thickness should be typically above 100 μm to cover the FP oscillations for a few periods. The sample dispersion and systematic noise can both result in a large TV value and affect the optimization. Scheller et al. proposed a more sensitive quasi-space (QS) method which demonstrated the capability for sub-100 μm samples [11], this method was also used more recently by Fastampa et al. to calibrate the pulse shift error in fiber-based TDS systems [12]. The principle is that the Fourier transform of a function with a periodic profile gives a large discrete peak. The thickness; thus, can be optimized by Fourier transforming *n* into the QS domain and minimizing the peak. The QS approach provides a better sensitivity. However, the versatility is still limited to achromatic transparent materials. The optical properties of chromatic dispersive materials such as water result in large QS values, which will be mixed with the QS values contributed from the FP effect and reduce the algorithm accuracy. Furthermore, when a sample has a molecular resonance in the spectrum such as lactose, both TV and QS methods are not applicable. Apart from the widely applied TV and QS methods, the work by Taschin et al. introduces another numerical thickness optimization method. The key principle is iteratively optimizing the optical properties at the best thickness achieved by smoothing the parameters in the last iteration, and it has been used to study ink thin films which were used in ancient manuscripts and drawings [13]. A similar method has also been reported by Yang et al. to optimize water thin-film thickness [14].

In this work, we propose a novel thickness optimization method based on fitting the material properties by a non-inflection function. The method is simple and fast with promising sensitivity and versatility. We will first analyze the theoretical foundation and the application principle of the method in Section 2. Three types of thin-film samples with very different optical properties were measured in Section 3 for the verification of the method. The achromatic and transparent dielectric PET (polyethylene terephthalate) films, the highly dispersive and absorptive water, and the lactose pallet which exhibits strong absorption peaks were all successfully characterized with an excellent accuracy on the thickness and the properties.

## 2. Method

**Figure 1:**
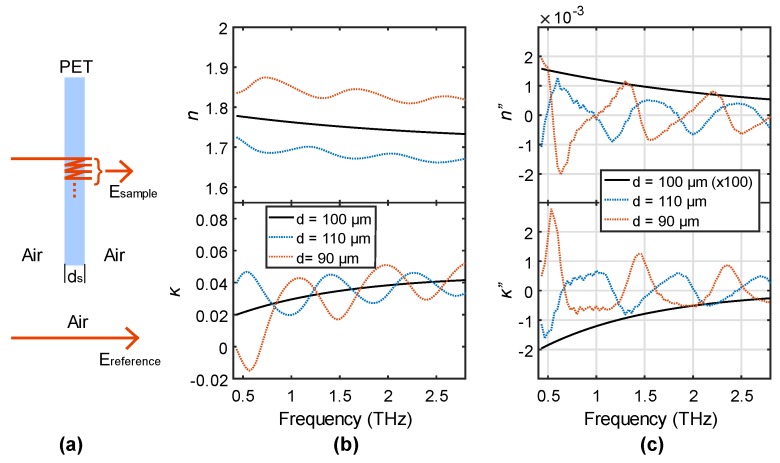
(**a**) Schematic of the transmission measurement of a PET (polyethylene terephthalate) thin film; (**b**) Extracted *n* and *κ* and (**c**) their second order derivatives when using the thickness of 100, 110, and 90 μm.

As mentioned above, characterizing a thin-film sample using an incorrect thickness usually results in FP oscillations in *n* and *κ*. We use a simple simulation to show this characteristic. Figure 1a shows a traditional process of thin-film transmission measurement (PET film in this example). In a measurement, a transmitted sample signal containing multiple internal reflections is measured, which is compared to the reference signal passing through air. Their ratio can be expressed as:

(1) $$\frac{E_{sample}\left(\omega \right)}{E_{reference}\left(\omega \right)}=\frac{t_{as}t_{sa}\exp\left(-i\omega d{\tilde{n}}_{s}/c\right)}{1+r_{as}r_{sa}\exp\left(-i2\omega d{\tilde{n}}_{s}/c\right)}\cdot exp\left(\frac{\omega d}{c}\right),$$

where *t*_as_ (*r*_as_) and *t*_sa_ (*r*_sa_) are the transmission (reflection) coefficients at the air-sample and sample-air interfaces, respectively. ${\tilde{n}}_{s}$ and *d* are the complex refractive index and the thickness of the sample, respectively. *c* is the speed of light. We assume the PET film in Figure 1a has the *n* and *κ* shown as the black curves in Figure 1b and a thickness of *d*_s_ = 100 μm. The sample-reference ratio can be calculated by the right part of the equation using these properties. We now simulate this ratio to be the experimental result in the left part of the equation, and we assume the sample properties are unknown and to be solved from the right part of Equation (1). As in this way we know the exact correct thickness, solving Equation (1) with *d* = 100 μm returns the sample *n* and *κ* we just used. Characterization using incorrect thicknesses of 110 and 90 μm results in the dotted blue and orange curves in Figure 1b. The 10% thickness error produces FP oscillations in both *n* and *κ* as expected. Here, we show the FP effect resulting from a wrong thickness can be sensitively recognized by the inflection points.

Visually, an inflection point is where the curve changes its curvature profile, such as from convex to concave, or vice versa. This can be clearly observed from the dotted blue and orange curves in Figure 1b, showing the periodic switch between these two curvature shapes, indicating that the inflection points are generated by the FP effect. Mathematically, an inflection point is a point of a continuous curve where its second derivative reaches zero and changes its signs. From this point of view, the periodic FP effect is similar to adding a sinusoidal function *sin*(x) to a slowly varied curve (the black curve) with an offset. The second derivative of *sin*(x) is proportional to *sin*(x) while the second derivative of a slowly varied function is very small. The sum of them is basically still a *sin* wave which contains many zero points (i.e., the inflection points). To verify this, we calculated the second derivatives of *n* and *κ* to evaluate the existence of inflection points in Figure 1c. The black curves have very small values and are magnified by a factor of 100, and the curves never cross with zero. On the contrary, the dotted blue and orange curves have large values and show the periodic sinusoidal profile, resulting in many zero values. In other words, the second derivatives obviously enhance the difference between using a correct and an incorrect thickness value, providing inflection points as a recognizable characteristic for the determination of the correct thickness.

The *n* and *κ* used for the above simulation were smoothed from the experimental data to make an ideal curve shape without noisy oscillations, which is the reason that the second derivative of the black curves is perfectly away from zero. In an actual measurement, noise must exist and propagates to the characterized *n* and *κ*. The actual property curves are not perfectly smooth and contain many random noisy oscillations. The corresponding second derivative will be frequently crossing zero to result in many fake inflection points. Therefore, the thickness cannot be determined by simply counting the inflection points. We propose a robust method by fitting *n* and *κ* to a non-inflection function and determine the optimized thickness from the best fit. The defined function should contain no inflection point and be able to describe the *n* and *κ* profile of most materials. As a result, the best fit can only be achieved when the correct thickness is given to remove the FP induced inflection points. Random oscillations from the noise have very little effect to the overall fitness. The simplest function satisfying the above requirement is a two-order (or lower orders) polynomial, *f*(*ω*) = *aω*^2^ + *bω* + *c*. The second derivative *f*’’(*ω*) = 2*a* indicates no inflection point. However, the two-order polynomial can only fit curves with a linear gradient change because *f*’(*ω*) = 2*aω* + *b* has a linear relationship with frequencies. This limits its adaptability. For example, the dielectric function of water is described by a double-Debye model, which has the first derivative obviously non-linear with frequencies [15]. Instead, an exponential function with an offset (referred to as the offset exponential function, or OE function herein), *f*(*ω*) = *a*exp(*bω*) + *c,* can better adapt to the profile of most materials. Any order derivative of this function is always an exponential function, which supports various curvature shapes to provide a great flexibility for the original function. The second derivative, which is also an exponential function, has no solution for *f’’*(*ω*) = 0 to prevent any inflection point. The major limitation of an OE function is its monotonic behavior (its first derivative is either always positive or always negative). However, from the wide range characterizations among crystals dielectrics and semiconductors [5], metal oxide [16], glass [7], polymer [6,8], polar liquids [4], and biomedical samples [17], we can see most of the materials have *n* and *κ* monotonously varied with frequencies in the THz range. In the very few cases that the monotonic condition is not satisfied, we can replace the constant offset term to a one-order polynomial, that is *f*(*ω*) = *a*exp(*bω*) + *cx + d*. This allows the first derivative to reach zero for fitting a non-monotonous property. 

## 3. Results

To experimentally verify the proposed method, we measured three different types of material. PET films with a reference thickness of 188, 125, and 50 μm were measured to demonstrate the application in low-dispersion low-loss material. Pure water, which is highly chromatic dispersive and absorptive, was sandwiched in between two sapphire wafers as a thin-film and characterized. We also processed data from a thin-film lactose pallet, which has several strong absorption peaks in the studied range, to show the great adaptability even to strongly frequency-resonant materials.

### 3.1. PET Thin Films

**Figure 2:**
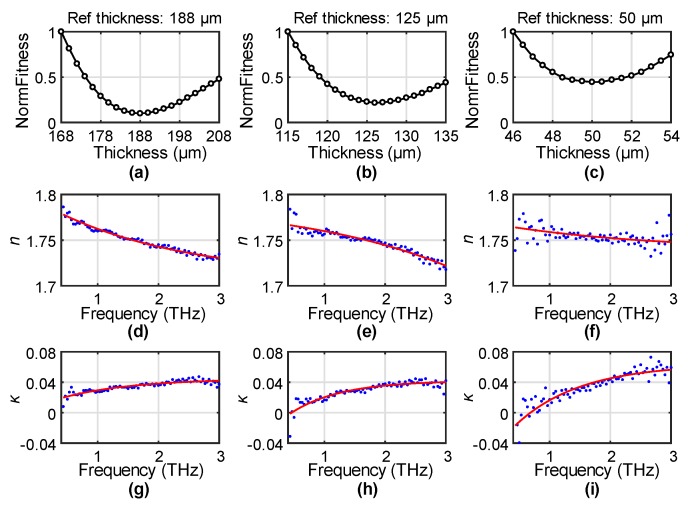
The normalized fitness (NormFitness) of the OE (offset exponential) functions to the extracted *n* (refractive index) and *κ* (extinction coefficient) for the (**a**) 188 μm, (**b**) 125 μm, and (**c**) 50 μm PET films. The experimental results of *n* for the (**d**) 188 μm, (**e**) 125 μm, and (**f**) 50 μm PET films and *κ* for the (**g**) 188 μm, (**h**) 125 μm, and (**i**) 50 μm PET films at their optimized thicknesses shown in the blue dots, with the red solid curves indicating the OE fitting.

PET is similar to many other polymers, which have low dispersion and low loss in the THz range [6]. Its high optical transparency, good surface smoothness (roughness rms less than a few nanometers) and simple fabrication of different thicknesses make it a popular eletro-optical material for various THz applications [18,19]. We measured three PET films with a reference thickness of 188 ± 5, 125 ± 5, and 50 ± 5 μm, respectively. The thickness was assumed to be unknown and was determined from the best fit to the OE function. In particular, different thicknesses around the reference values were inputted to extract the *n* and *κ* for each sample. *n* and *κ* were then fitted individually by the OE function. The fitting root-mean-square error (RMSE) of *n* and *κ* were summed up to represent the fitness of a specific thickness. The normalized fitness (normalize the largest value to be 1) of the three samples are shown in Figure 2a–c, respectively.

The NormFitness results show a high sensitivity to the thickness from 188 to 50 μm. The minimum values in Figure 2a–c are 188, 126, and 50 μm, respectively. Using a 5% error for the NormFitness, the corresponding uncertainties of the optimized thicknesses in Figure 2a–c are ±1, ±1.5, and ±1 μm, respectively. These results are almost perfectly the same as their reference values. The thinnest film of 50 μm can still provide a high contrast in the fitness value with a single minimum, indicating the robust performance on sub-100 μm samples. *n* results, characterized by using the optimized thickness of the 188, 126, and 50 μm, are shown as the blue dots in Figure 2d–f, respectively, with their corresponding *κ* values shown as the blue dots in Figure 2g–i. The red curves in these figures give the corresponding best-fitted OE functions. A high-degree match was found for all the results, which demonstrates the capability of the proposed OE function for precisely describing PET properties under the correct thickness. The best fitting was achieved for the thickest sample, while the thinner samples had a relatively larger fitting error. This is because thinner samples provide less contrast to the transmission signal, making the characterization result more sensitive to the measurement errors. This can also be confirmed from the larger errors at lower frequencies as the thickness is more obviously smaller than the wavelength.

**Figure 3:**
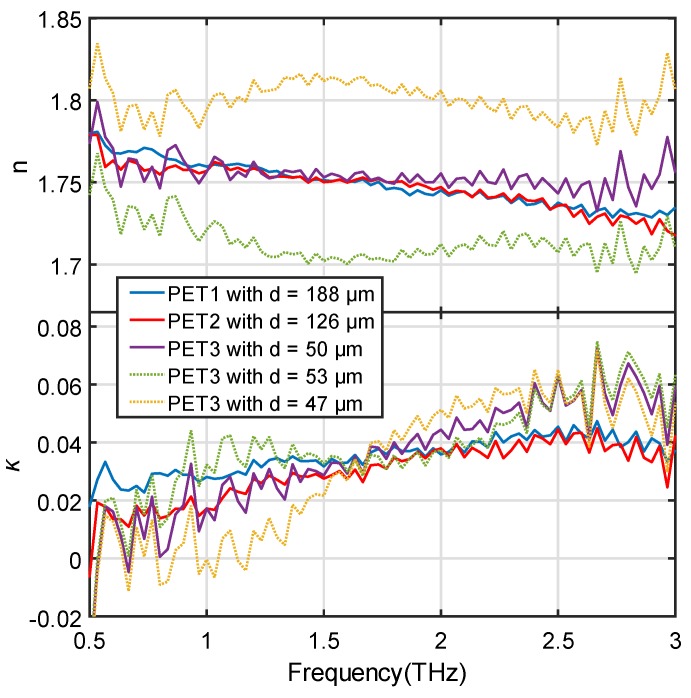
*n* and *κ* of the 188 μm (PET1), 125 μm (PET2), and 50 μm (PET3) PET films at their optimized thicknesses (solid curves), compared to the *n* and *κ* of PET3 characterized by using the optimized thickness with a ±3 μm error.

We also compare the *n* and *κ* of the three samples by the optimized thickness in Figure 3. This provides an efficient way to evaluate the accuracy of the optimized thickness. Only when all the three optimized thickness values are correct can their *n* and *κ* all match with each other. PET1, PET2, and PET3 in the figure legends correspond to the PET samples with the reference thickness of 188, 125, and 50 μm. They were characterized by the corresponding thickness *d* in the legend. The solid curves give the result by the optimized thickness values. All the three curves match very well with each other for both *n* and *κ.* Using PET1 as a reference, the average difference in *n* and *κ* for the PET2 are 0.0063 and 0.0073, and for PET3 are 0.0164 and 0.0139. All the samples show no observable periodic FP oscillations. We also added ±3 μm to the optimized thickness of PET3. The corresponding *n* and *κ* are shown in the dotted curves. As expected, the added thickness error shifts *n* away from the results of the solid curves with slightly noticeable FP oscillations. The corresponding *κ* values also produces some change in the curvature shape between concave and convex, which will not allow good fitting by the OE function. These comparisons comprehensively prove that the thicknesses optimized from the fitting are very close to their actual thicknesses.

### 3.2. Thin-Film Water

In contrast to polymers, water is highly absorptive and chromatic dispersive in the THz range. The high absorption requires the sample to be prepared as an ultra-thin film to maintain the transmitted signal intensity, typically no more than 200 μm. Liquid samples are usually measured in a thin-film liquid cell, which is a sandwich structure with two windows of known optical properties and a spacer of a known thickness value. However, the actual liquid thickness is often different from the spacer reference thickness, due to potential imperfect contact between the spacer and the windows. Thin film spacers are also soft and their thickness is related to the pressure applied to assemble the cell. These result in a large thickness uncertainty in water measurement. Additionally, the large dispersion results in large TV or QS values in the previous method, which cannot be distinguished from the FP effect. Another reported numerical method for estimating the water thickness is by using the Kramers-Kronig relations [20]. This technique requires a relatively broad bandwidth that has a relatively low sensitivity.

**Figure 4:**
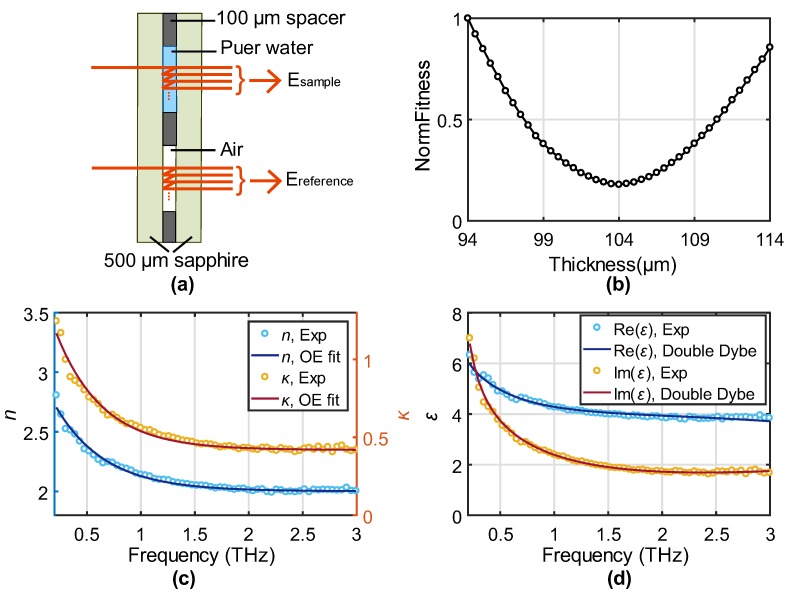
(**a**) Schematic of the water thin film measurement. (**b**) NormFitness as a function of thickness ranging for 94–114 μm. (**c**) *n* and *κ* of water extracted from the optimized thickness (open circles) and the fitting to the OE functions (solid curves). (**d**) Complex permittivity of water at the optimized thickness (open circles) and the fitting to the double-Debye model.

Using OE fitting elegantly overcomes this difficulty. Here, we sandwiched a water thin film in between two 500 μm sapphire windows by a 100 μm spacer, as shown in Figure 4a. The spacer has an uncertainty of ±2 μm. However, as mentioned above, the contact situation is a more important factor for the thickness uncertainty and can introduce a thickness error over a few micrometers. The signals transmitted through the water and the empty cell were detected and compared for sample characterizations according to the three-layer Fresnel model [1], with the refractive of 3.06−0.002i used for the C-cut sapphire [5]. Thicknesses ranging from 94 to 114 μm were sent for parameter extraction, and OE fitting was applied to the resulting *n* and *κ,* respectively. The normalized fitness as a function of thickness was shown in Figure 4b. The best thickness was found at 104 μm, which is close but slightly different from the spacer thickness. The sensitivity of the fitness on the thickness is good and shows a good adaptability to highly absorptive and dispersive materials. The extracted *n* and *κ* by the optimized thickness are shown in the light blue and orange open circles in Figure 4c, respectively. The dark blue and red solid curves indicate the best fit by the OE function, showing a high-degree match. This also verifies our analysis in the Method section that the OE function can well describe dispersive properties, while a two-order polynomial cannot provide a good fitting (the gradient from 1.5 to 3 THz is almost zero, while the gradient at low frequencies is very large). The results are highly consistent with published work by Jepsen et al. and Soltani et al. [21,22]. 

To verify the accuracy of the optimized thickness, we use the double-Debye model to fit the extracted complex permittivity. Considering the frequency range studied, which covers up to 3 THz, a stretching vibration term at around 5.3 THz was added [23,24]. The model is expressed as [23]:

(2) $$\varepsilon \left(\omega \right)=\varepsilon_{\infty }+\frac{\Delta \varepsilon_{1}}{1+i\omega \tau_{1}}+\frac{\Delta \varepsilon_{2}}{1+i\omega \tau_{2}}+\frac{A_{s}}{\omega_{s}^{2}-\omega^{2}+i\omega \gamma_{s}},$$

where the first term is the permittivity at infinite frequency, the second and third terms represent the slow and fast Debye relaxations, and the last term is contributed by the stretching mode. Considering the precise measurement at the microwave frequencies (where the model becomes a single-Debye model) and the weak contribution from the high-frequency vibration, we adopted *τ*_1_ =9.4 ps, *ω*_s_/2π = 5.3 THz, and *γ*_s_/2π = 5.35 THz from Yada’s work [23]. The other parameters were extracted from the best fitting, with *ε*_∞_ = 2.5 (2.0, 2.5), Δ*ε*_1_ = 77.9 (74.9, 74.9), Δ*ε*_2_ = 1.94 (1.67, 2.8), *τ*_2_ = 0.299 ps (0.25 ps, 0.3 ps), and *A*_s_ = 1486 THz (1244 THz, 1500 THz). The values in the brackets are from [23] and [24], respectively. These results give a reasonable physical description and are in good agreement with the literature. The fitting to the real and imaginary part of the permittivity was plotted in Figure 4d, showing a high-degree match to the experimental result. The successful double-Debye modeling verifies the accurate thickness optimized from the OE fitting from the physical point of view.

### 3.3. Thin-Film Lactose Pallet

Lactose exhibits strong absorption peaks in the THz range, with the most obvious two peaks centered at 0.53 and 1.37 THz [25,26]. Similar to the water measurement, the high absorption requires a sufficiently thin sample to reduce the signal attenuation. To well resolve the lactose absorption peaks, the pallet should be thinner than 400 μm. Pellets below 500 μm are typically very fragile, making the thickness measurement very difficult and inaccurate. The absorption lines result in dense echoes in the time-domain, which fully mix with the multiple reflections, thus it is also impossible to determine the thickness from the separated pulses. Furthermore, the existence of these absorption peaks introduces highly frequency-dependent optical properties, making none of the reported numerical methods applicable for thickness determination. We show the high flexibility and adaptability of this OE fitting method to lactose sample by simply fitting to the two non-absorption peak regions in the refractive index.

**Figure 5:**
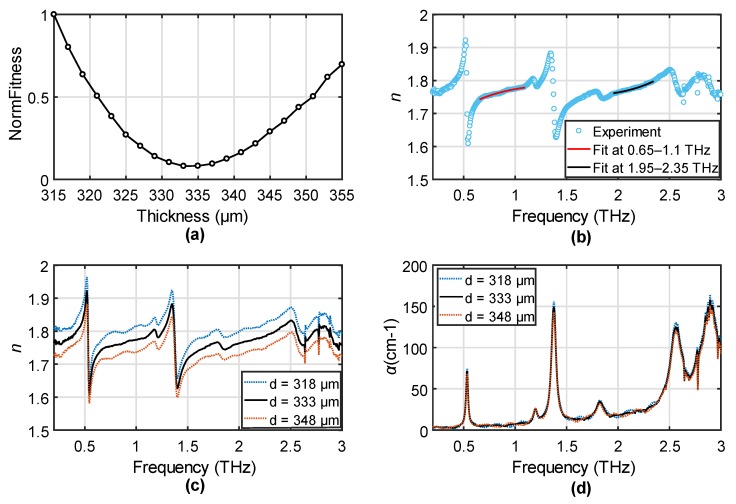
(**a**) NormFitness as a function of thickness for the lactose pallet. (**b**) The extracted refractive index and the optimized thickness and the fitting to the OE functions in the range 0.65–1.1 THz and 1.95–2.35 THz. (**c**) *n* and (**d**) *α* of the lactose pallet extracted by using the optimized thickness (black solid curve) and the optimized thickness with a ±15 μm error (dotted curves).

A thin lactose pellet was measured in transmission similar to the steps for the PET film measurement. Due to the fragility, we can only roughly measure the thickness by a caliper to be around 350 μm. The thickness uncertainty of the caliper is ±10 μm, while the uncertainty due to the imperfect contact between the caliper and the fragile lactose could be larger. To determine the actual thickness, we fit the OE function to the refractive index in the range 0.65–1.1 THz and 1.95–2.35 THz, respectively. We did not fit the extinction ratio because the absorptions generate concave shapes between two absorption peaks for the extinction ratio, which cannot be well fitted by the OE function. The normalized fitness values to the thickness range 315–355 μm is shown in Figure 5a. The optimized thickness value was found at 333 μm, which is 17 μm smaller than the physical measurement. The fitting results to the refractive index are shown in Figure 5b. The light blue circles show the experimental result by using the thickness of 333 μm, and the red and black solid curves indicate the fitting at the two defined regions. The good match is achieved due to the absence of FP oscillations. To confirm this, the refractive index and absorption coefficient (*α*) by using the optimized thickness are shown in Figure 5c,d, respectively, compared with the results by using the optimized thickness adding with a ±15 μm variation shown in the dotted curves. Although due to the relatively small refractive index and the high absorption, the multiple reflections are relatively weak, we can still confirm the existence of the FP oscillations in the dotted curves by comparing to the black curve. The thickness error also changes the absorption amplitude, which may affect the modeling accuracy of the peaks [27]. The use of the OE fitting method greatly reduces the thickness uncertainty to improve the accuracy, which is the first method being successfully applied to the thickness determination of materials with strong absorption fingerprints.

## 4. Discussion

In this work, we theoretically show that material properties characterized from an incorrect thickness value contain FP oscillations, which are mathematically represented by the generated inflection points. On the contrary, an inflection point seldom exists when the correct thickness is given. Based on this, we propose a thickness determination method by fitting the material *n* and *κ* to an OE function. The OE function is very robust to the curvature shape and is able describe the optical properties of most materials, while containing no inflection point to disallow the existence of FP effect. In this way, the optimized thickness can be determined from the best fitting. Experimental measurements on three different types of sample were performed to demonstrate the versatility and high sensitivity of the method. The experimental results show that materials of low and high absorption, achromatic and dispersive, featureless or containing resonant absorptions, can all be well fitted, with thickness ranging from tens to hundreds of micrometers being accurately determined. To highlight the improvement of our approach over existing techniques, we have adopted the reported TV and QS approaches to process the above three types of samples and showed their performance in Appendix A. The comparison quantitatively demonstrates the superior sensitivity, accuracy, and versatility of the proposed OE fitting method over the state-of-art.

**Figure A2:**
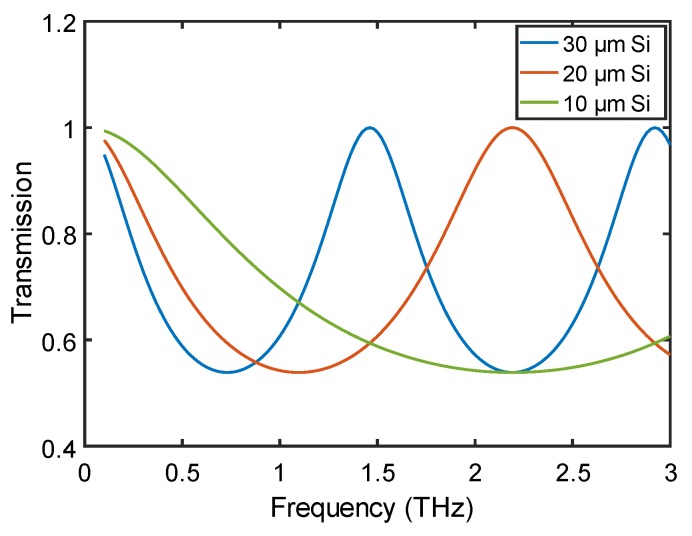
Simulated transmission spectrum of Si thin film with different thicknesses.

According to the principle, the minimum thickness limitation that can be determined from the method theoretically depends on the refractive index of the material and the system bandwidth. As long as the effective spectrum can cover over half of the FP period, at least one inflection point can be detected. For example, measuring a silicon thin film in a 3 THz bandwidth system, the minimum thickness could be less than 10 μm (see Figure A2 in Appendix B), which is comparable or even better than the state-of-art [11]. Practically, the noise influence and the intensity of the FP effect would also affect the accuracy. For example, a very thin film of low refractive index provides very little contrast to the THz waves, thus the extracted properties may be very noisy to well resolve the FP oscillations. The FP intensity should also be sufficiently large to result in a bad fitting when an incorrect thickness is given. For example, measuring a 200 μm water thin film has almost no FP effect due to the high absorption, while measuring thin-film ethanol in between polymer spacers loses the FP effect due to the index matching. In this case, none of the reported numerical methods would be applicable either, as all of them are based on FP effect detection.

In summary, the proposed method demonstrates superior sensitivity compared to reported numerical algorithms. It also has the best versatility that it is applicable for highly-absorptive and highly-dispersive thin films. Furthermore, materials with significant absorption features can also be well characterized. The demonstrated robustness and versatility will benefit a variety of research areas and industrial applications, such as bio-tissue imaging, time-of-flight studies, as well as paper or car-coating thickness determination.

## Figures and Tables

**Figure A1:**
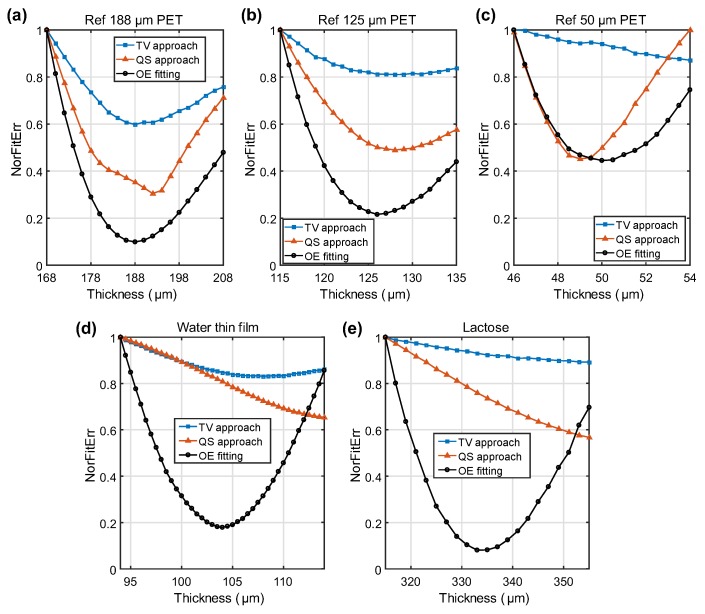
The *NorFitErr* (Normalized Fitness Error) as a function of thickness. The minimum values correspond to the optimized thickness found from different techniques. The results of the PET thin film with a reference thickness of (**a**) 188 μm, (**b**) 125 μm, and (**c**) 50 μm, and the results of the (**d**) water and (**e**) lactose thin film by the TV (Total Variation), QS (Quasi-Sapce), and OE fitting methods are shown for a comparison.

## Appendix A

To quantitatively compare the performance of the proposed OE fitting method with existing techniques, we applied the TV approach and QS approach to our measurement data. The TV and QS approaches evaluate the refractive index and/or the extinction coefficients of the PET (three thicknesses), water, and lactose samples to optimize the sample thickness [2,11]. In detail, the TV values are acquired by summing up the absolute differences of all the neighboring refractive indices and extinction ratios:

$$TV=\sum \left|n_{m-1}-n_{m}\right|+\left|\kappa_{m-1}-\kappa_{m}\right|.$$

The QS values are acquired by Fourier transforming the refractive index and extracting the corresponding discrete peak values. The corresponding fitting error (TV values in the TV approach and QS values in the QS approach) are normalized to the largest value in the corresponding thickness range and labeled as *NorFitErr*. The results are shown in Figure A1.

The TV approach shows a relatively good accuracy to the 188 μm PET film, but starts losing its sensitivity for the 125 μm PET sample, as the *NorFitErr* is almost flat from 125 to 132 μm. Consistent with the previous analysis that the limitation of TV approach is usually above 100 μm, it is unable to optimize the thickness of the 50 μm sample due to the low sensitivity. The TV optimization to the water data also has a very low sensitivity and the minimum value can only be roughly determined between 105 and 110 μm. The QS approach has a better sensitivity as introduced, but it also has a higher requirement on the sample achromatic property because the dispersion could contribute to large QS values. The accuracy and the sensitivity for the slightly-dispersive PET films is acceptable but not as good as the OE fitting method (by comparing to the reference thickness and the good match between the three samples using the optimized thicknesses from the OE method in Figure 3). This is mainly due to the large QS contribution from the small dispersion of the PET film. On the contrary, the QS approach is unable to process the water data due to the strong dispersion. For the lactose which exhibits absorption features, both TV and QS approaches are not applicable. The comparison demonstrates the superior performance of the OE fitting method in sensitivity, accuracy, and versatility over the existing TV and QS approaches.

## Appendix B

Figure A2 shows the simulated transmission spectrum of Si thin film with thicknesses of 30, 20, and 10 μm, respectively. We can see the 10 μm curve can still cover over half of the FP period, resulting in one inflection point (at around 0.9 THz) being able to be possibly detected.

## References

[1] Fujiwara H. (2007). Spectroscopic Ellipsometry Principles and Applications.

[2] Dorney T.D., Baraniuk R.G., Mittleman D.M. (2001). Material parameter estimation with terahertz time-domain spectroscopy. Opt. Soc. Am..

[3] Withayachumnankul W., Fischer B.M., Lin H., Abbott D. (2008). Uncertainty in terahertz time-domain spectroscopy measurement. J. Opt. Soc. Am. B.

[4] Kindt J.T., Schmuttenmaer C.A. (1996). Far-Infrared Dielectric Properties of Polar Liquids Probed by Femtosecond Terahertz Pulse Spectroscopy. J. Phys. Chem..

[5] Grischkowsky D., Keiding S., van Exter M., Fattinger C. (1990). Far-infrared time-domain spectroscopy with terahertz beams of dielectrics and semiconductors. J. Opt. Soc. Am. B.

[6] Jin Y., Kim G., Jeon S. (2006). Terahertz Dielectric Properties of Polymers. J. Korean Phys. Soc..

[7] Naftaly M., Miles R.E. (2007). Terahertz Time-Domain Spectroscopy for Material Characterization. Proc. IEEE.

[8] Cunningham P.D., Valdes N.N., Vallejo F.A., Hayden L.M., Polishak B., Zhou X.H., Luo J., Jen A.K.Y., Williams J.C., Twieg R.J. (2011). Broadband terahertz characterization of the refractive index and absorption of some important polymeric and organic electro-optic materials. J. Appl. Phys..

[9] Pupeza I., Wilk R., Koch M. (2007). Highly accurate optical material parameter determination with THz time-domain spectroscopy. Opt. Express.

[10] Duvillaret L., Garet F., Coutaz J.-L. (1999). Highly precise determination of optical constants and sample thickness in terahertz time-domain spectroscopy. Appl. Opt..

[11] Scheller M., Jansen C., Koch M. (2009). Analyzing sub-100-μm samples with transmission terahertz time domain spectroscopy. Opt. Commun..

[12] Fastampa R., Pilozzi L., Missori M. (2017). Cancellation of Fabry-Perot interference effects in terahertz time-domain spectroscopy of optically thin samples. Phys. Rev. A.

[13] Taschin A., Bartolini P., Tasseva J., Torre R. (2018). THz time-domain spectroscopic investigations of thin films. Meas. J. Int. Meas. Confed..

[14] Yang F., Liu L.P., Song M.J., Zhang F. (2018). Fast Determination of Optical Constants and Sample Thickness of Thin Liquid Samples in Terahertz Time-Domain Spectroscopy. J. Appl. Spectrosc..

[15] Rønne C., Thrane L., Åstrand P.O., Wallqvist A., Mikkelsen K.V., Keiding S.R. (1997). Investigation of the temperature dependence of dielectric relaxation in liquid water by THz reflection spectroscopy and molecular dynamics simulation. J. Chem. Phys..

[16] Chen X., Parrott E.P.J., Huang Z., Chan H., Pickwell-MacPherson E. (2018). Robust and Accurate Terahertz Time-Domain Spectroscopic Ellipsometry. Photonics Res..

[17] He Y., Liu K., Au C., Sun Q., Parrott E.P.J., Pickwell-MacPherson E. (2017). Determination of terahertz permittivity of dehydrated biological samples. Phys. Med. Biol..

[18] Markelz A.G., Roitberg A., Heilweil E.J. (2000). Pulsed terahertz spectroscopy of DNA, bovine serum albumin and collagen between 0.1 and 2.0 THz. Chem. Phys. Lett..

[19] Liu Z., Huang C.-Y., Liu H., Zhang X., Lee C. (2013). Resonance enhancement of terahertz metamaterials by liquid crystals/indium tin oxide interfaces. Opt. Express.

[20] Son H., Choi D.-H., Park G.-S. (2017). Improved thickness estimation of liquid water using Kramers–Kronig relations for determination of precise optical parameters in terahertz transmission spectroscopy. Opt. Express.

[21] Jepsen P.U., Møller U., Merbold H. (2007). Investigation of aqueous alcohol and sugar solutions with reflection terahertz time-domain spectroscopy. Opt. Express.

[22] Soltani A., Jahn D., Duschek L., Castro-Camus E., Koch M., Withayachumnankul W. (2015). Attenuated total reflection terahertz time-domain spectroscopy: uncertainty analysis and reduction scheme. IEEE Trans. Terahertz Sci. Technol..

[23] Yada H., Nagai M., Tanaka K. (2008). Origin of the fast relaxation component of water and heavy water revealed by terahertz time-domain attenuated total reflection spectroscopy. Chem. Phys. Lett..

[24] Nagai M., Yada H., Arikawa T., Tanaka K. (2006). Terahertz time-domain attenuated total reflection spectroscopy in water and biological solution. Int. J. Infrared Millimeter Waves.

[25] Fischer B., Hoffmann M., Helm H., Modjesch G., Jepsen P.U. (2005). Chemical recognition in terahertz time-domain spectroscopy and imaging. Semicond. Sci. Technol..

[26] Bjarnason J.E., Brown E.R., Korter T.M. (2007). Comparison of the THz absorption feature in lactose to related saccharides. Terahertz Mil. Secur. Appl. V.

[27] Brown E.R., Fedor J.E.B.M., Korter T.M. (2011). On the strong and narrow absorption signature in lactose at 0.53 THz. Appl. Phys. Lett..

